# Experimental Validation of Alzheimer's Disease Targets Prioritized Through Systems Biology

**DOI:** 10.1002/alz70859_103181

**Published:** 2025-12-25

**Authors:** Gregory A. Cary

**Affiliations:** ^1^ The Jackson Laboratory, Bar Harbor, ME USA

## Abstract

**Background:**

Genome‐wide association studies and multi‐omic analyses of Alzheimer's disease have generated extensive lists of potential therapeutic targets. Effectively prioritizing these targets for drug development remains a significant challenge. To address this, we have developed a platform to articulate interpretable hypotheses based on systems‐level signatures of risk for AD patient data. Using this platform we prioritized hypotheses including mitochondrial hypometabolism and chronic neuroinflammation. However, it remains unclear which predicted driver genes are capable of affecting the targeted biology in appropriate cellular contexts.

**Method:**

This study presents an approach to rapidly filter prioritized candidate proteins to those that can robustly modify targeted biological functions *in vitro*. We used mouse BV2 cells as a microglial surrogate and included cells in which the Psen2 gene is stably knocked down as an additional cell state. Each cell line was transfected with a panel of siRNA targeting the 29 candidate genes and phenotypes were assessed. We measured phenotypes relevant to the nominating hypotheses including Mitotracker TMRM and Alamar Blue to assess mitochondrial functions and pHrodo Green Zymosan Bioparticles and transcriptional output from NF‐κB using an luminescent reporter to assess immune functions. We also assessed the proteomic effects of knockdown for each target.

**Result:**

Several targets impacted cellular phenotypes and we focused on those with corresponding changes in proteomic signatures to ensure robust effects. We identify targets that reverse key proteomic signatures associated with disease. We also note the importance of including sensitizing cell conditions (i.e. Psen2 knockdown) as these experiments yielded more phenotypes and altered proteomic responses to target perturbation. This analysis revealed several promising targets – Pdhb, Pdha1, Dlat, Ap2a2, and Psmc3 – which significantly modulated both phenotypes and proteomic profiles.

**Conclusion:**

Our approach is able to filter large sets of putative driver genes nominated from integrated systems‐level analyses of patient data to those capable of impacting the targeted biological processes. The TREAT‐AD consortium will prioritize further resource development for targets validated by these experiments.

